# Analysis on the Radiation Property of the Bounded Modes of Periodic Leaky-Wave Structure with Finite-Length Using a Hybrid Method

**DOI:** 10.1038/srep22917

**Published:** 2016-03-18

**Authors:** Zheng Li, Junhong Wang, Jianjie Duan, Zhan Zhang, Meie Chen

**Affiliations:** 1 Key Laboratory of All Optical Network & Advanced Telecommunication Network of MOE, Beijing Jiaotong University, Beijing, 100044, China; 2 Institute of Lightwave Technology, Beijing Jiaotong University, Beijing, 100044, China

## Abstract

In this paper the radiation property of the one-dimensional periodic leaky-wave structure is analysed using a new hybrid method, which involves the mode expansion method for expanding the periodic aperture field in terms of spatial harmonics and the method of effective radiation sections for transforming the expanded fields into far fields. Using this method, the radiation of each spatial harmonic can be achieved, and the contributions of the harmonics (especially the bounded modes) to the total radiation of the periodic leaky-wave structure can be calculated. The main findings in this paper demonstrate that the bounded modes in a finite length structure have obvious contribution to the far-field radiation, which was considered to be non-radiative and always ignored in the conventional researches.

In general, there are two types of leaky-wave radiation structures. The first one is the uniform leaky-wave structures, such as the rectangular waveguide with long axial slot which can be equivalent to a linear magnetic current^1^. The second one is the periodic leaky-wave structures, such as the rectangular waveguide with periodic slots^2^, slit metal waveguide with periodic grooves^3^, periodic dielectric waveguide^4^, and composite right/left-handed leaky-wave antennas with very small period^5^,^6^. For the periodic structures, according to Floquet theorem, periodic field distributions will be generated on the structure apertures, which can further be seen as the superposition of infinite spatial harmonics^7^,^8^. When one or more of these harmonics have axial propagation constants less than that in environment, then they become the radiating modes (fast waves) and travel away from the structure; the other spatial harmonics will attenuate exponentially in the transverse direction, and can only propagate along the structure, which are known as the bounded modes (slow waves)^8^,^9^. So for radiation property analysis of infinitely long periodic leaky-wave structures, only the radiating modes need to be considered. For periodic leaky-wave structures with finite length, the same method is usually adopted to calculate far field, and the bounded modes are not taken into account^2^,^9^. However, in this paper it will be shown that for the periodic leaky-wave structures with finite length, the bounded modes have obvious contribution to the total far-field radiation, which should not be ignored.

The bounded modes involved in a periodic aperture field can be seen as a series of uniform slow waves (ignoring attenuation). The traditional viewpoint about slow-wave radiation is that the radiation can only occur at terminals of the finite structure^10^,^11^. In our previous work^12^,^13^, the radiation mechanism of the slow-wave structure was presented from a new perspective, which indicated that the radiation of the uniform traveling-wave structures is generated by a pair of Effective Radiation Sections (ERSs), locating close to the ends of the structure. Therefore, ERSs can be an efficient method for calculating the radiation field of the uniform traveling-wave structure with finite length. It is worth noting that the ERS method is suitable for all types of traveling-wave structures (either slow- or fast-wave structures). It is briefly presented in the supplementary information at the end of this paper. However, the ERS method has never been used in periodic structures. In this paper, the application of the ERS method is extended to the periodic leaky-wave structures. Since the aperture field of the periodic structure can be regarded as a superposition of infinite spatial harmonics, and each harmonic is a traveling wave, so the ERS method can be employed for calculating the radiation by each harmonic.

In order to extract the propagation constant of each spatial harmonic from the periodic aperture field, mode expansion must be implemented. In^14^, a mode expansion method was presented in cylinder coordinate which is suitable for far field calculation of leaky coaxial cable. In this paper, a new mode expansion method in Cartesian coordinate is presented. Combining this method with the ERS method, a hybrid approach is achieved, which is efficient for studying the radiation property of one-dimensional periodic leaky-wave structure. Using this method we can find the fact that the bounded waves also have contributions to the total radiation field, which was considered to be non-radiative in the conventional research.

## Results

### Mode Expansion Method for the Aperture Field of One-Dimensional Periodic Structure

In this paper, only the one-dimensional periodic leaky-wave structures are considered. The leaky rectangular waveguide (LRWG) with periodic transverse slots is a typical type of periodic leaky-wave structure, and can be regarded as one-dimensional periodic structure. Although it is not an ideal one-dimensional structure due to the finite width and field variation in the transverse direction, the proposed method will prove effective for this structure.

**Figure 1:**
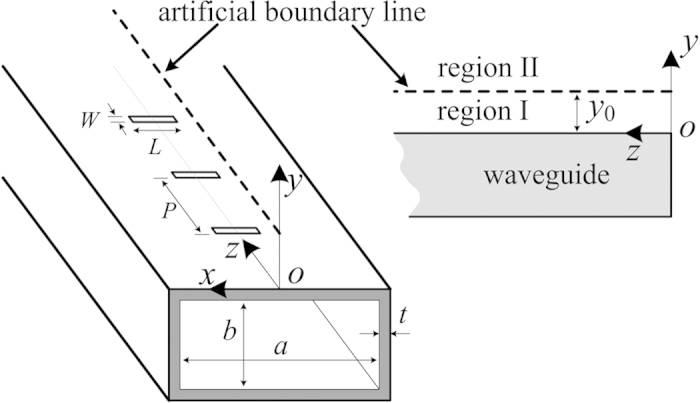
The geometry of the LRWG.

The LRWG, which is widely used in the communication-based train control (CBTC) system^15^, is shown in Fig. 1. Its radiation property in *yoz* plane (vertical plane passing through the axis of waveguide) will be studied (one-dimension approximation). An artificial boundary line in *yoz* plane is set above the top of LRWG with height of *y*_0_, which separates the upper half space of *yoz* plane into two regions (named region I and region II). The waveguide is considered to be made by perfect electric conductor (PEC), and the leakage loss is small due to small slot and large period, so the attenuation of aperture field along the artificial line is very small, which is ignored in the preliminary study for simplicity.

The fields in region I denoted by *E*^I^ is calculated by the full-wave method. The fields in region II can be expressed by *z* components of the electric and magnetic Hertzian vector potentials *Π*_*e*_ and *Π*_*m*_ as


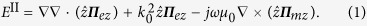


Since the field in region II is periodic in *z* direction, *Π*_*ez*_ and *Π*_*mz*_ can be expanded into Floquet’s modes according to the Floquet theorem as following


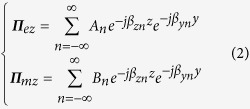


where *A*_*n*_ and *B*_*n*_ are unknown coefficients to be determined, and *β*_*zn*_ and *β*_*yn*_ are the propagation constants of the *n*th spatial harmonic in *z* and *y* directions respectively. *β*_*zn*_ and *β*_*yn*_ can be expressed as


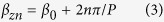



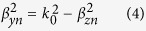


where *β*_0_ is the propagation constant of the fundamental mode (*n* = 0), and *k*_0_ is the propagation constant in free space.

Substituting (2) into (1), the fields in region II can be obtained as following


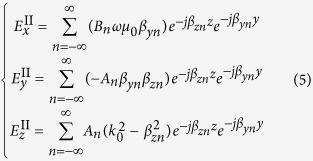


The tangential components of the fields must be continuous on the artificial boundary. Since the component *E*_*x*_ is zero along the boundary line (due to symmetry), only the component *E*_*z*_ needs to be considered, that is


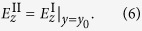


**Figure 2:**
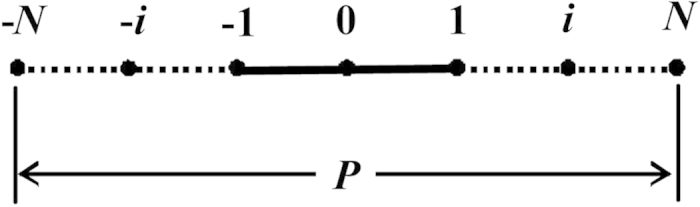
Nodes along the aperture within one period.

The length in one period along the artificial boundary line is discretized into (2*N* + 1) nodes uniformly in the *z* direction, and the sequence number of each node is denoted by *i*, as shown in Fig. 2.

Using equation (6) at each node (point matching), we obtain equation (7) as


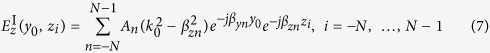


In equation (7), the summation has been confined within *n*∈[−*N, N* − 1]. Thus, 2*N* equations can be obtained from (7), and the number of unknowns is also 2*N*. Therefore, a unique solution can be achieved. The truncation error due to the limited summation in (7) can be neglected if *N* is large enough. Equation (7) can be written in matrix forms as


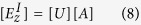


where the matrix elements are


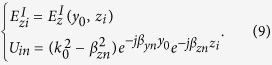


Then the coefficient matrix [*A*] can be derived, and hence each spatial harmonic is determined.

It is worth noting that this method is suitable for arbitrary multi-harmonic radiation. However, the LRWG usually works under the condition of mono-harmonic radiation. So in this work the mono-harmonic radiation condition is required to ensure the existence of only one radiating mode. Also, this makes it simple to demonstrate the contributions of the bounded modes to the far field radiation, since all the other spatial harmonics (except the sole radiating mode) will be the bounded modes. The mono-harmonic radiation condition requires that:


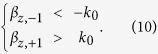


Substituting (3) into (10), we get the mono-harmonic radiation condition:


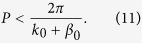


### Mode Expansion Results

The LRWG used in this paper (as shown in Fig. 1) is based on the standard waveguide WR430 with internal dimensions *a* = 109.22 mm, *b* = 54.61 mm, and thickness *t* = 2.03 mm. The parameters of periodical transverse slots are: *W* = 3 mm, *L* = 19 mm, *P* = 61 mm. The whole structure has 15 unit cells, so the total length is 915 mm. This LRWG operates at 2.4 GHz. The propagation constant of the fundamental mode (*n* = 0) *β*_0_ is calculated by full-wave simulation of the aperture field phase at different slots, and we get *β*_*z*,0_ = *β*_0_ = 41.30 rad/m. Thus, according to equation (11), the mono-harmonic radiation condition is *P* < 68.62 mm, so the current period length of *P* = 61 mm meets the requirement.

**Figure 3:**
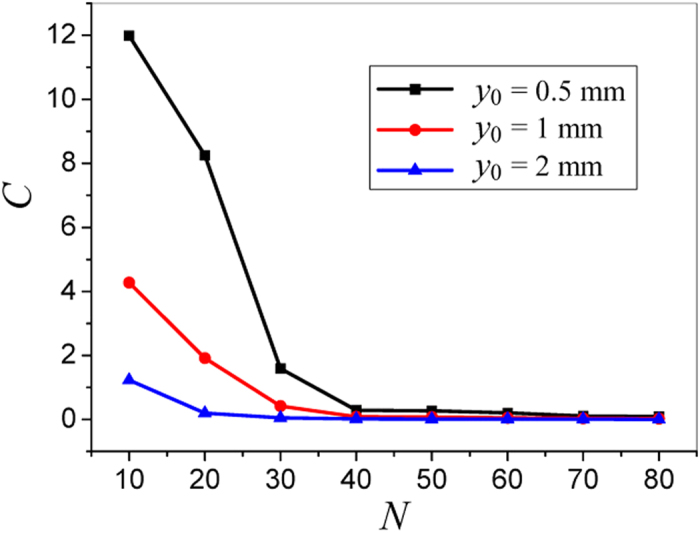
The variation of the evaluating function for different harmonics order *N* and the height of the equivalent line.

The computational accuracy of the proposed method depends on the value of *N* in equation (7). In order to find a proper *N* that ensures enough accuracy, an evaluating function is defined as


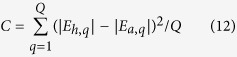


where *E*_*a*_ is the simulated aperture field in one period, *E*_*h*_ is the summation of fields of the harmonics in one period, and *Q* is the total number of the simulated field points in one period. The convergent curves of this function for different height *y*_0_ are illustrated in Fig. 3. It is found that for larger *y*_0_, convergence of (12) becomes faster, and smaller *N* can give enough accuracy. This is because that the field closer to the LRWG surface contains more contributions from the higher order harmonics. If the number of terms in equation (7) is insufficient, the contributions of higher order harmonics are not sufficiently involved. Therefore, according to Fig. 3, the value of *N* is set to be 50 for the case of *y*_0_ = 0.5 mm in the following study.

**Figure 4:**
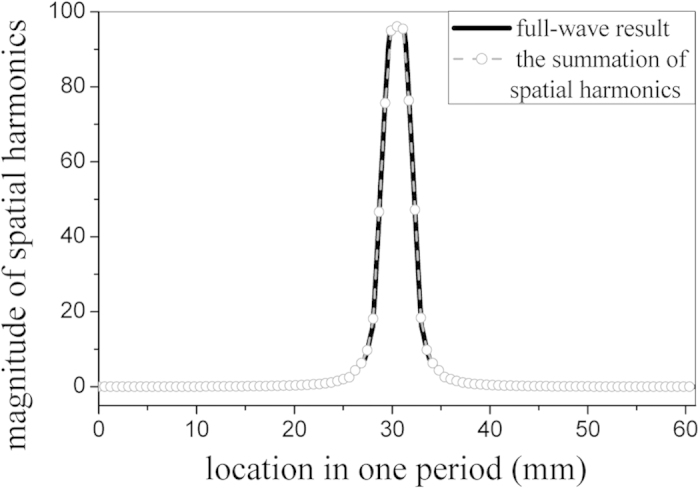
Comparison of the simulated aperture field and the summation of all the expanded spatial harmonics (in one period when *N *= 50).

**Figure 5:**
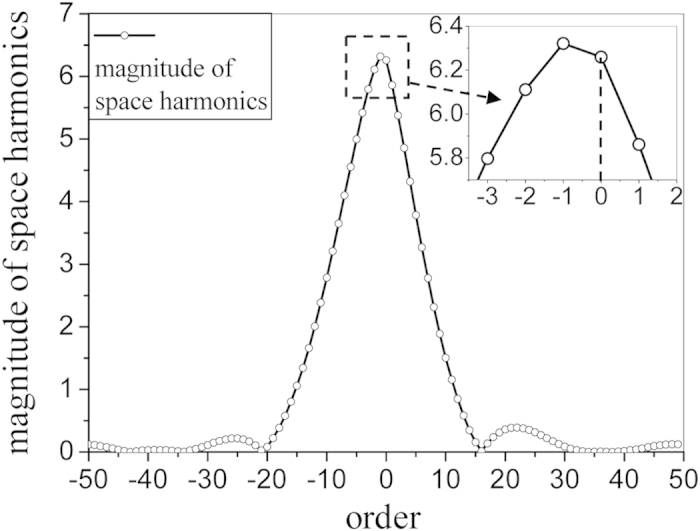
The magnitudes of the expanded spatial harmonics.

The simulated result of the aperture field in one period is given in Fig. 4, and it is expanded into different spatial harmonics using the mode expansion method (equations (8) and (9)). Figure 5 illustrates the magnitudes of the spatial harmonics. It can be seen that the magnitude of *n* = −1 harmonic is greater than that of the *n* = 0 harmonic. This is caused by the periodic perturbation along the structure, yielding that the *n* = −1 harmonic possesses more power than the *n* = 0 harmonic. When *N* = 50, the summation of all the spatial harmonics obtained using equation (7) is also given in Fig. 4 for comparison, which shows a good match with the simulated result.

### Radiations of the Harmonics Calculated by ERSs

The method and whole procedure to calculate the radiation of periodic aperture field by using ERSs are stated as following: Each spatial harmonic extracted from the periodic field can be viewed as a traveling wave, and the radiation characteristic can be analysed using ERS method. The determination of ERSs and the formulae for calculating far field by ERSs were given in the supplementary information according to the study in^13^. So the far fields either from radiating harmonics or from the bounded harmonics can be calculated. Then the far-field radiation of the whole periodic structure can be expressed as the summation of radiation fields from all the spatial harmonics as following


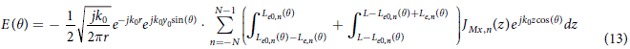


where *L*_*e*0_ and *L*_*e*_ are the location and length of the ERS. *r* is the distance from observation point to origin. *J*_*Mx*_ is the equivalent magnetic current for each spatial harmonic. The two terms in (13) represents the magnetic current integrations along the two sections of ERSs, respectively.

**Figure 6:**
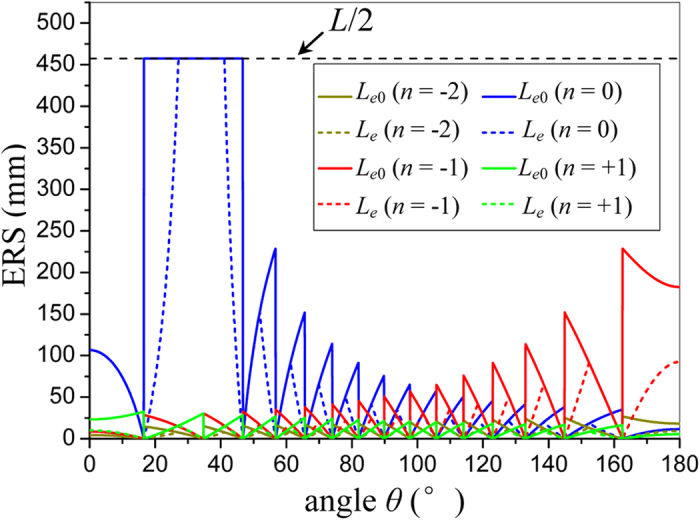
ERSs of different spatial harmonics as functions of angle *θ*.

So firstly, we calculate the ERSs of each spatial harmonic by supplementary formulas (S4) and (S5) in the supplementary information, and the results are shown in Fig. 6. It can be seen that for the fundamental mode (*n* = 0), the ERSs corresponding to the main beam direction (around *θ* = 34.8°) cover the whole length of the LRWG, demonstrating that the far field in the main beam direction is generated by the whole structure. For the *n* = −1 spatial harmonic, the ERSs are very short at first, but increases rapidly as *θ* increases. When *θ* enters the angular range corresponding to the last several lobes close to *θ* = 180°, the ERSs of the *n* = −1 spatial harmonic is found to be much larger than that of the *n* = 0 spatial harmonic. Predictably, this will lead to a much stronger radiation in this angular range generated by the *n* = −1 spatial harmonic, though it is actually a bounded mode. For the *n* = +1 and −2 spatial harmonics, for any angle in space, the corresponding ERSs are very short, which means a weak radiation. It is worth noting that different spatial harmonics have different locations and lengths of ERSs, due to different propagation constants.

**Figure 7:**
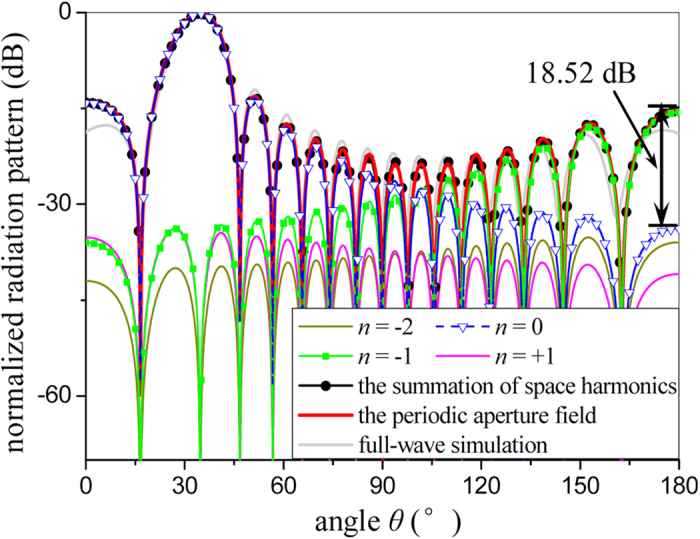
Comparison of the normalized radiation patterns of each spatial harmonic, the summation of all harmonics, and the three dimensional full-wave simulation of the whole LRWG, respectively.

Checking each term of the summation in equation (13) individually, the radiation pattern of each spatial harmonic can be calculated by the corresponding ERSs. Figure 7 shows the normalized radiation patterns of each spatial harmonic and the summation of all harmonics, respectively, and the radiation pattern calculated from the simulated aperture field is also given for comparison. It is observed that the radiation patterns generated by the fundamental mode (*n* = 0, the radiating mode, blue curve with triangles) and the simulated aperture field (red curve) match well with each other within *θ*∈[0°, 65°]. However, as *θ* increases, the difference between the two curves becomes significant, and reaches 18.52 dB on the last lobe. Contrarily, when *θ* increases, the radiation pattern of *n* = −1 harmonic (green curve) approaches to the radiation pattern calculated by the aperture field gradually, and agrees with it on the last several lobes. This demonstrates that the last several lobes generated by the aperture field are mainly contributed by the radiation of *n* = −1 harmonic. Although the *n* = −1 spatial harmonic is a bounded mode, its effect on the total radiation cannot be ignored, which can be also inferred from the larger ERSs in Fig. 6. In addition, in Fig. 7 the radiation strengths of *n* = −2 and *n* =  +1 spatial harmonics are relatively weak, corresponding to the smaller ERSs in Fig. 6. In Fig. 7, the black curve with dots (the summation of all the spatial harmonics) is in good agreement with the red curve (direct radiation of the aperture field), because the contributions of higher order harmonics (bounded waves) have been sufficiently involved when *N* = 50. In addition, the radiation patterns of the whole LRWG obtained by the three-dimensional full-wave simulation (CST) is also given in Fig. 7 (grey curve), from which we find that the radiation pattern calculated directly from the aperture field of one-dimension model is very close to that of three-dimensional full-wave method. This means that the proposed method is valid for the analysis of this type of periodic leaky-wave structure.

**Figure 8:**
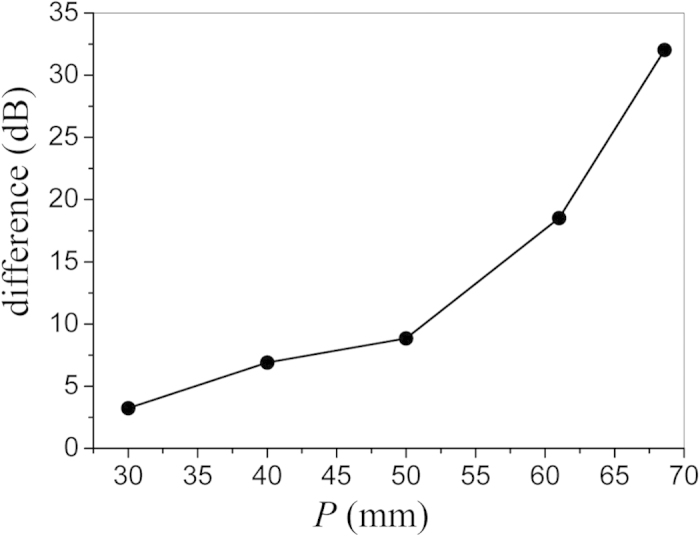
The difference of the last lobe levels generated by the fundamental mode (*n *= 0) and the total aperture field, respectively.

Figure 8 illustrates the difference between the last lobe levels obtained by the fundamental mode (*n* = 0) and the total aperture field, respectively. It can be seen that the difference becomes larger as *P* increases, and reaches 32 dB when *P* is 68.6 mm (this value of *P* is the critical value of the mono-harmonic condition). Therefore, the radiation property of this periodic structure is dominated by the fundamental mode only when the period of the slots is small, even when the mono-harmonic condition is satisfied. In addition, the period of the slots is the determination factor for the main beam direction, which is very important in practical applications of waveguide slot array. Therefore, proper period of slots should be chosen through a comprehensive analysis.

## Discussion

In this paper, taking the LRWG as an example, the radiation property of one-dimensional periodic leaky-wave structure is studied with a new hybrid method, which involves the mode expansion method and the ERSs method. Using this method, the periodic aperture field of the LRWG can be efficiently expanded into spatial harmonics. Then the radiating mode and bounded modes can be easily extracted from the total radiation field, and the contribution of each mode can be calculated using the ERSs of each spatial harmonics. The results show that the *n* = −1 spatial harmonic, which was usually supposed to be the non-radiating mode (slow wave) in the conventional research, has great influence on the total far-field radiation, yielding an obvious difference between the radiation patterns of the fundamental mode (*n* = 0) and the total periodic aperture field. The maximum difference of the last lobe levels can reach 32 dB even under the condition of mono-harmonic radiation. Therefore, the contribution of the bounded waves to the far-field radiation should not be ignored if high accuracy is demanded. Although the bounded waves contribute mainly to the higher order side lobes, the discovery in this paper changes the conventional opinion that the bounded mode (slow wave) can only be guided along the waveguide and do not have any contribution to the far fields. This is the most important discovery in this paper. From another perspective, this work presents a new insight into the mechanism of high side lobe levels generated by bounded-wave radiation, and this might be instructive for further research on the suppression of bounded-wave radiation.

## Methods

The study in this paper is based on a newly proposed hybrid method, which involves the mode expansion method for expanding the periodic aperture field in terms of spatial harmonics and the method of ERSs for transforming the expanded fields into far fields. In addition, full-wave simulation is executed to obtain the real aperture field and far field pattern using the commercial simulation software CST.

## Additional Information

**How to cite this article**: Li, Z. *et al*. Analysis on the Radiation Property of the Bounded Modes of Periodic Leaky-Wave Structure with Finite-Length Using a Hybrid Method. *Sci. Rep.* **6**, 22917; doi: 10.1038/srep22917 (2016).

## Supplementary Material

Supplementary Information

Supplementary Information
