# Supplementary material for: Analysis on the Radiation Property of the Bounded Modes of Periodic Leaky-Wave Structure with Finite-Length Using a Hybrid Method
Source: Sci Rep. 2016 Mar 18;6:22917. doi: 10.1038/srep22917 (PMC4796814; doi:10.1038/srep22917)
Supplement: Supplementary Information [file srep22917-s1.pdf]

# Analysis on the Radiation Property of the Bounded Modes of Periodic Leaky-Wave Structure with Finite-Length Using a Hybrid Method

**Authors: Zheng Li*, Junhong Wang, Jianjie Duan, Zhan Zhang, and Meie Chen**

## Supplementary Information: Method of Effective Radiation Section

The ERS method can be explained using Supplementary Fig. S1, in which a line-source model is given, representing a uniform traveling-wave structure (the total length is *L*)[13].

**Supplementary Figure S1: A line-source model of traveling-wave structure.**

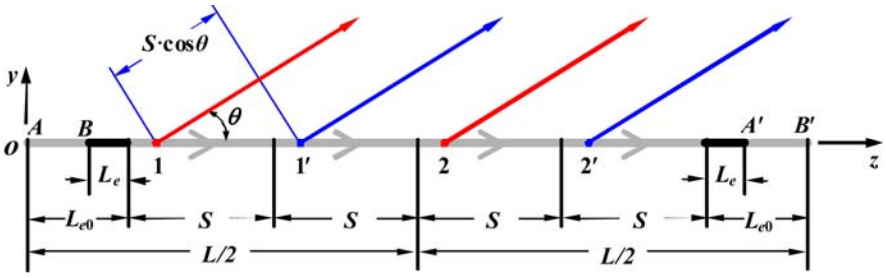


Firstly assuming that the wave attenuation when propagating along the traveling-wave structure is zero ($\alpha$ = 0). At a given observation angle $\theta$, if the emissions from points 1 and 1′ with distance *S* satisfy the following equation

$$k_0 S\cos\theta - \beta S = \pm\pi \tag{S1}$$

Then the emissions from two successive segments (the length is *S*) will be cancelled out completely in far-field region. The length *S* is defined by

$$S(\theta) = \pi / \left|k_0(\cos\theta - \beta_z / k_0)\right|, \tag{S2}$$

Also, if the fields from points *A* and *A*′, *B* and *B*′ at the given angle $\theta$ in the far-field region satisfy the following equation

$$k_0 S\cos\theta - \beta S = \pm(2N+1)\pi\,, \tag{S3}$$

then the far-field radiation from section *AB* can be cancelled by that from *A*′*B*′ completely, and only two short sections with length $L_e$ (named Effective Radiation Sections, ERSs, the black sections in Supplementary Fig. S1) need to be considered when calculating the radiation from the whole traveling-wave structure. In Supplementary Fig. S1 the length $L_{e0}$ is defined by

$$L_{e0}(\theta) = \left(L - 2N(\theta)S(\theta)\right)/2\,, \qquad (\,L_{e0}(\theta) \le L/2\ \ ) \tag{S4}$$

where *N* represents the number of segment pairs with spacing *S*. The length $L_e$ is defined by

$$L_e(\theta) = \begin{cases} L_{e0}(\theta) & \text{, when } L_{e0}(\theta) \le S(\theta)/2 \\ S(\theta) - L_{e0}(\theta), & \text{when } L_{e0}(\theta) > S(\theta)/2 \end{cases}. \tag{S5}$$

In the above equations, *N*, *S*, $L_{e0}$ are functions of angle $\theta$. Then the radiation pattern of the whole traveling-wave structure can be expressed as

$$f(\theta) = \sin\theta \cdot \left[ \left( \int_{L_{e0}(\theta)-L_e(\theta)}^{L_{e0}(\theta)} + \int_{L-L_{e0}(\theta)}^{L-L_{e0}(\theta)+L_e(\theta)} \right) e^{-j\beta y} e^{jk_0 y\cos\theta} dy \right], \tag{S6}$$

Furthermore, if the attenuation constant $\alpha$ along the structure is considered, the radiation pattern of the whole traveling-wave structure will be expressed as

$$f(\theta) = \cos\theta \cdot \int_0^L e^{-(j\beta+\alpha)y} e^{jk_0 y \sin\theta} dy$$

$$= \cos\theta \cdot \left[ \left( \int_{L_{e0}(\theta)-L_e(\theta)}^{L_{e0}(\theta)} + \int_{L-L_{e0}(\theta)}^{L-L_{e0}(\theta)+L_e(\theta)} \right) e^{-(j\beta+\alpha)y} e^{jk_0 y \sin\theta} dy \right] + \cos\theta \cdot \left[ \left( \int_0^{L_{e0}(\theta)-L_e(\theta)} + \int_{L-L_{e0}(\theta)+L_e(\theta)}^{L} + \int_{L_{e0}(\theta)}^{L-L_{e0}(\theta)} \right) e^{-(j\beta+\alpha)y} e^{jk_0 y \sin\theta} dy \right] . \quad (S7)$$

For common leaky-wave and surface-wave structures, if the attenuation constant $\alpha$ along the structure is small, equation (S6) can usually meet the demand of calculation accuracy according to the research in [12, 13].
