# Supplementary material for: Analysis on the Radiation Property of the Bounded Modes of Periodic Leaky-Wave Structure with Finite-Length Using a Hybrid Method
Source: Sci Rep. 2016 Mar 18;6:22917. doi: 10.1038/srep22917 (PMC4796814; doi:10.1038/srep22917)
Supplement: Supplementary Information [file srep22917-s2.doc]

**Analysis on the Radiation Property of the Bounded Modes of Periodic Leaky-Wave Structure with Finite-Length Using a Hybrid Method**

**Authors: Zheng Li**^*^**, Junhong Wang, Jianjie Duan, Zhan Zhang, and Meie Chen**

**Supplementary Information: Method of Effective Radiation Section**

The ERS method can be explained using Supplementary Fig. S1, in which a line-source model is given, representing a uniform traveling-wave structure (the total length is *L*)^13^.

**Supplementary Figure S1: A line-source model of traveling-wave structure.**


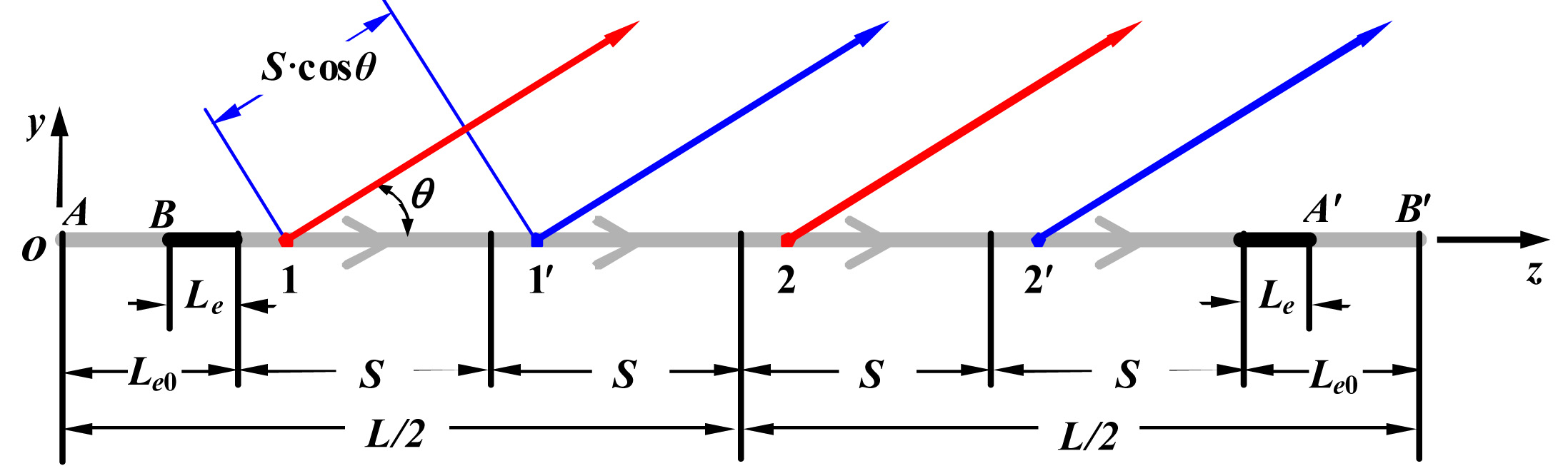


Firstly assuming that the wave attenuation when propagating along the traveling-wave structure is zero (*α* = 0). At a given observation angle *θ*, if the emissions from points 1 and 1′ with distance *S* satisfy the following equation

$k_{0}S\cos\theta-\beta S=\pm\pi$ (S1)

Then the emissions from two successive segments (the length is *S*) will be cancelled out completely in far-field region. The length *S* is defined by

$S(\theta)=\pi/\left|k_{0}(\cos\theta-\beta_{z}/k_{0})\right|$, (S2)

Also, if the fields from points *A* and *A*′, *B* and *B*′ at the given angle *θ* in the far-field region satisfy the following equation

$k_{0}S\cos\theta-\beta S=\pm\left(2N+1\right)\pi$, (S3)

then the far-field radiation from section *AB* can be cancelled by that from *A*′*B*′ completely, and only two short sections with length *L_e_* (named Effective Radiation Sections, ERSs, the black sections in Supplementary Fig. S1) need to be considered when calculating the radiation from the whole traveling-wave structure. In Supplementary Fig. S1 the length *L_e_*_0_ is defined by

$L_{e0}(\theta)=\left(L-2N(\theta)S(\theta)\right)/2$, ($L_{e0}(\theta)\leq L/2$ ) (S4)

where *N* represents the number of segment pairs with spacing *S*. The length *L_e_* is defined by

$L_{e}(\theta)=\left\{\begin{matrix} L_{e0}(\theta)\text{ , when }L_{e0}(\theta)\leq S(\theta)/2 \\ S(\theta)-L_{e0}(\theta)\text{, when }L_{e0}(\theta)>S(\theta)/2 \end{matrix}\right.$. (S5)

In the above equations, *N*, *S*, *L_e_*_0_ are functions of angle *θ*. Then the radiation pattern of the whole traveling-wave structure can be expressed as

$f\left(\theta\right)=\sin\theta\cdot\left[(\int_{L_{e0}(\theta)-L_{e}(\theta)}^{L_{e0}(\theta)} +\int_{L-L_{e0}(\theta)}^{L-L_{e0}(\theta)+L_{e}(\theta)} )e^{-j\beta y}e^{jk_{0}y\cos\theta}dy\right]$, (S6)

Furthermore, if the attenuation constant *α* along the structure is considered, the radiation pattern of the whole traveling-wave structure will be expressed as

$$f\left(\theta\right)=\cos\theta\cdot\int_{0}^{L} e^{-\left(j\beta+\alpha\right)y}e^{jk_{0}y\sin\theta}dy$$

$=\cos\theta\cdot\left[(\int_{L_{e0}(\theta)-L_{e}(\theta)}^{L_{e0}(\theta)} +\int_{L-L_{e0}(\theta)}^{L-L_{e0}(\theta)+L_{e}(\theta)} )e^{-\left(j\beta+\alpha\right)y}e^{jk_{0}y\sin\theta}dy\right]+\cos\theta\cdot\left[(\int_{0}^{L_{e0}(\theta)-L_{e}(\theta)} +\int_{L-L_{e0}(\theta)+L_{e}(\theta)}^{L} +\int_{L_{e0}(\theta)}^{L-L_{e0}(\theta)} )e^{-\left(j\beta+\alpha\right)y}e^{jk_{0}y\sin\theta}dy\right]$.( S7)

For common leaky-wave and surface-wave structures, if the attenuation constant *α* along the structure is small, equation (S6) can usually meet the demand of calculation accuracy according to the research in [12, 13].
